# Butyrate decreases its own oxidation in colorectal cancer cells through inhibition of histone deacetylases

**DOI:** 10.18632/oncotarget.25546

**Published:** 2018-06-05

**Authors:** Anna Han, Natalie Bennett, Bettaieb Ahmed, Jay Whelan, Dallas R. Donohoe

**Affiliations:** ^1^ Department of Nutrition, University of Tennessee, Knoxville, TN 37996, USA

**Keywords:** butyrate, HDAC inhibitor, SCAD, β-oxidation, metaboloepigenetics

## Abstract

Colorectal cancer is characterized by an increase in the utilization of glucose and a diminishment in the oxidation of butyrate, which is a short chain fatty acid. In colorectal cancer cells, butyrate inhibits histone deacetylases to increase the expression of genes that slow the cell cycle and induce apoptosis. Understanding the mechanisms that contribute to the metabolic shift away from butyrate oxidation in cancer cells is important in in understanding the beneficial effects of the molecule toward colorectal cancer. Here, we demonstrate that butyrate decreased its own oxidation in cancerous colonocytes. Butyrate lowered the expression of short chain acyl-CoA dehydrogenase, an enzyme that mediates the oxidation of short-chain fatty acids. Butyrate does not alter short chain acyl-CoA dehydrogenase levels in non-cancerous colonocytes. Trichostatin A, a structurally unrelated inhibitor of histone deacetylases, and propionate also decreased the level of short chain acyl-CoA dehydrogenase, which alluded to inhibition of histone deacetylases as a part of the mechanism. Knockdown of histone deacetylase isoform 1, but not isoform 2 or 3, inhibited the ability of butyrate to decrease short chain acyl-CoA dehydrogenase expression. This work identifies a mechanism by which butyrate selective targets colorectal cancer cells to reduce its own metabolism.

## INTRODUCTION

Colorectal cancer (CRC) is the third most common and lethal cancer in the United States [1]. Diet is one of the strongest influential risk factors in the development of colorectal cancer. Thus, dietary intervention has been suggested to be an effective way to decrease colorectal cancer development and mortality [2–4]. Many studies have observed a beneficial or protective effect toward colorectal cancer, although other studies have reported contradicted these findings [5–8]. The fermentation of dietary fiber in the proximal colon produces bacterial derived-short chain fatty acids (SCFAs), which include acetate, propionate and butyrate. Among these SCFAs, butyrate has been considered a critical metabolite that mediates the tumor repressive effect of dietary fiber toward colorectal cancer [9–11].

Unlike other SCFAs, butyrate is primarily metabolized by colonocytes as an energy source and also plays a role in epigenetic modification where it increases histone acetylation through inhibiting histone deacetylases [12–14]. At physiologically relevant doses, butyrate decreases cell proliferation, increases cell differentiation and induces apoptosis in colorectal cancer cells [15–17]. Interestingly, butyrate’s effects on cell proliferation and apoptosis are associated with its metabolic fate in colonocytes [18, 19]. Several studies have shown that butyrate is primarily oxidized by isolated colonocytes, and that this oxidation can be influenced by exogenous factors [20–22]. Additionally, butyrate has been shown to decrease proliferation in cancerous colonocytes and increase proliferation in non-cancerous colonocytes [21, 23–25]. Thus, butyrate produces different effects on cancerous and normal colonocytes, which have different metabolic characteristics. In normal colonocytes, butyrate is oxidized through mitochondrial β-oxidation and is utilized to produce energy through the tricarboxylic acid (TCA) cycle or cytosolic acetyl-CoA. The acetyl-CoA produced in this process can also be used as a cofactor for histone acetyltransferases (HATs) or as a substrate for lipogenesis [18, 26]. However, cancerous colonocytes favor glucose over butyrate utilization as a result of a metabolic transformation called the Warburg effect. This glucose-preferred environment results in suppressed butyrate oxidation, and enhanced histone acetylation induced by butyrate [18, 19]. Therefore, to understand butyrate’s inhibitory and selective effects against colorectal cancer, it is essential to understand the mechanisms that contribute to decreased butyrate oxidation in cancerous colonocytes.

Short chain acyl-CoA dehydrogenase (SCAD) is an enzyme that catalyzes the first step in the oxidation of butyrate in the mitochondria [27]. Deletion of short chain acyl-CoA dehydrogenase in humans and mice decreases butyrate oxidation whiles simultaneously increasing butyryl-CoA accumulation and excretion, thereby illustrating the enzyme’s role in butyrate metabolism [28–30]. Additionally, loss of SCAD in isolated colonocytes results in a decrease in the oxidation of butyrate [31]. Previous studies have observed a reduction in SCAD expression at both gene and protein level in colorectal cancer [32–35]. However, there little is known about the mechanisms that regulate SCAD expression. In this study, we report that butyrate decreases its own metabolism in colorectal cancer cells. Pretreatment of colorectal cancer cells with butyrate suppresses SCAD expression. The mechanism as to how butyrate lowers SCAD levels in colorectal cancer cells is explored and is mediated through inhibition of a histone deacetylase. Importantly, non-cancerous colonocytes do not show this effect. This work identifies butyrate itself as part of the mechanism by which colorectal cancer cells shift away from utilizing butyrate.

## RESULTS

### Butyrate diminishes its own oxidation in colorectal cancer cells

**Figure 1:**
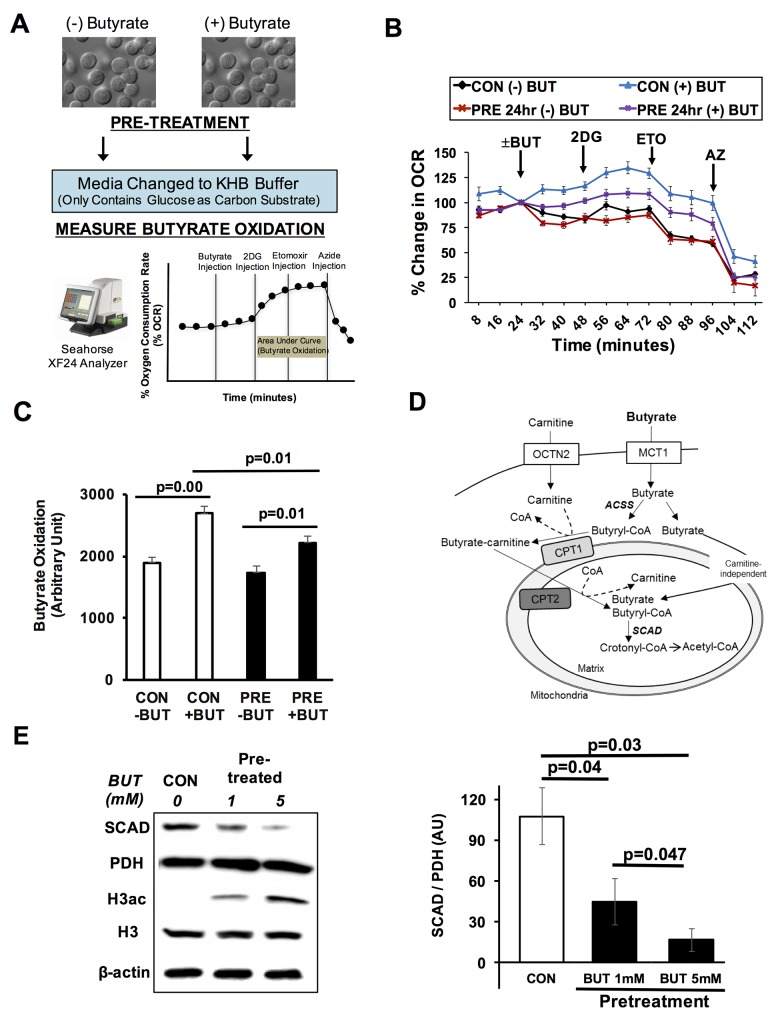
Butyrate suppresses its own oxidation in colorectal cancer cells **(A)** Schematic shows butyrate pre-pretreatment and measurement of butyrate oxidation. **(B)** Percentage change in oxygen consumption rate (OCR) relative to baseline in pre-treated HCT116 cells with and without butyrate (5 mM, 24 hrs). **(C)** Area under the curve measurement from OCR analysis taken after 2-deoxyglucose (2DG) injection but before azide injection (56-104 min). **(D)** Diagram of butyrate oxidation in the cancerous colonocytes. Cancerous colonocytes oxidize butyrate in the mitochondria. SCAD plays a role in first step of the oxidation of butyrate. **(E)** Western blot confirming butyrate reduced the SCAD level. Quantification of western blots is shown in right panel. Data for western blots and butyrate oxidation represent the average of 3-5 replicates per condition. Error bars are ± SEM. MCT1; monocarboxylate transport protein 1, OCTN2; organic cation/carnitine transporter, ACSS; Acyl Co-A synthetases, CPT1/2; carnitine palmitoyltransferase 1/2, SCAD; short chain acyl-CoA dehydrogenase.

In recent years, our lab has sought to identify factors or conditions that impact the oxidation of butyrate in colorectal cancer cells. Since butyrate modulates gene expression through inhibiting histone deacetylases, we postulated that butyrate could directly affect its own metabolic fate through changing the expression of proteins that oxidize short chain fatty acids. To begin to test this possibility, HCT116 colorectal cancer cells were pretreated with or without butyrate, and butyrate oxidation was measured as the percent change in the oxygen consumption rate (Figure 1A). In the assay, cells are maintained in a Fatty Acid Oxidation Media containing KHB buffer and only 2.5 mM glucose as the only exogenous carbon substrate. During the assay butyrate, 2-deoxyglucose (2DG), etomoxir, and sodium azide are injected into the media. The 2DG blocks glucose utilization, thus leaving butyrate as the sole exogenous energetic substrate. Etomoxir, inhibits carnitine palmitoyltransferase and the contribution of endogenous lipids to oxidative metabolism. Butyrate oxidation represents the total area under the curve following the addition of 2DG to the time when sodium azide is injected. In pretreated cells, butyrate repressed its own oxidation when compared to non-pretreated cells (Figure 1B). After 2DG injection, butyrate oxidation was significantly lower in pretreated cells as compared to non-pretreated control cells (Figure 1C). RKO cells were used to confirm whether butyrate pretreatment would decrease butyrate oxidation in an additional colorectal cancer line. Indeed, pretreatment with butyrate in RKO cells significantly diminished butyrate oxidation as compared to cells not pretreated with butyrate (Supplementary Figure 1). This suggested that the ability of butyrate to suppress its own oxidation is not limited to a single colorectal cancer cell line.

After butyrate moves into the mitochondria, short chain acyl-CoA dehydrogenase (SCAD) catalyzes the first dehydrogenation step where butyryl-CoA is metabolized to form acetyl-CoA [27] (Figure 1D). Western blot analysis of HCT116 colorectal cancer cells showed that butyrate treatment decreased the levels of SCAD (Figure 1E). Butyrate dose-dependently lowered the expression of SCAD. Since butyrate is known to increase global histone acetylation, H3 acetylation was also measured to confirm the cells were responding to butyrate as expected. Butyrate dose-dependently increasing histone H3 acetylation. Throughout this paper (unless otherwise mentioned), pyruvate dehydrogenase (PDH) is used as a loading control instead of β-Actin due to the fact that PDH is also a mitochondrial protein like SCAD. Moreover, by normalizing with PDH, it separates changes in SCAD expression from alterations in mitochondrial number.

### Butyrate pretreatment does not affect its own oxidation in isolated colonocytes

**Figure 2:**
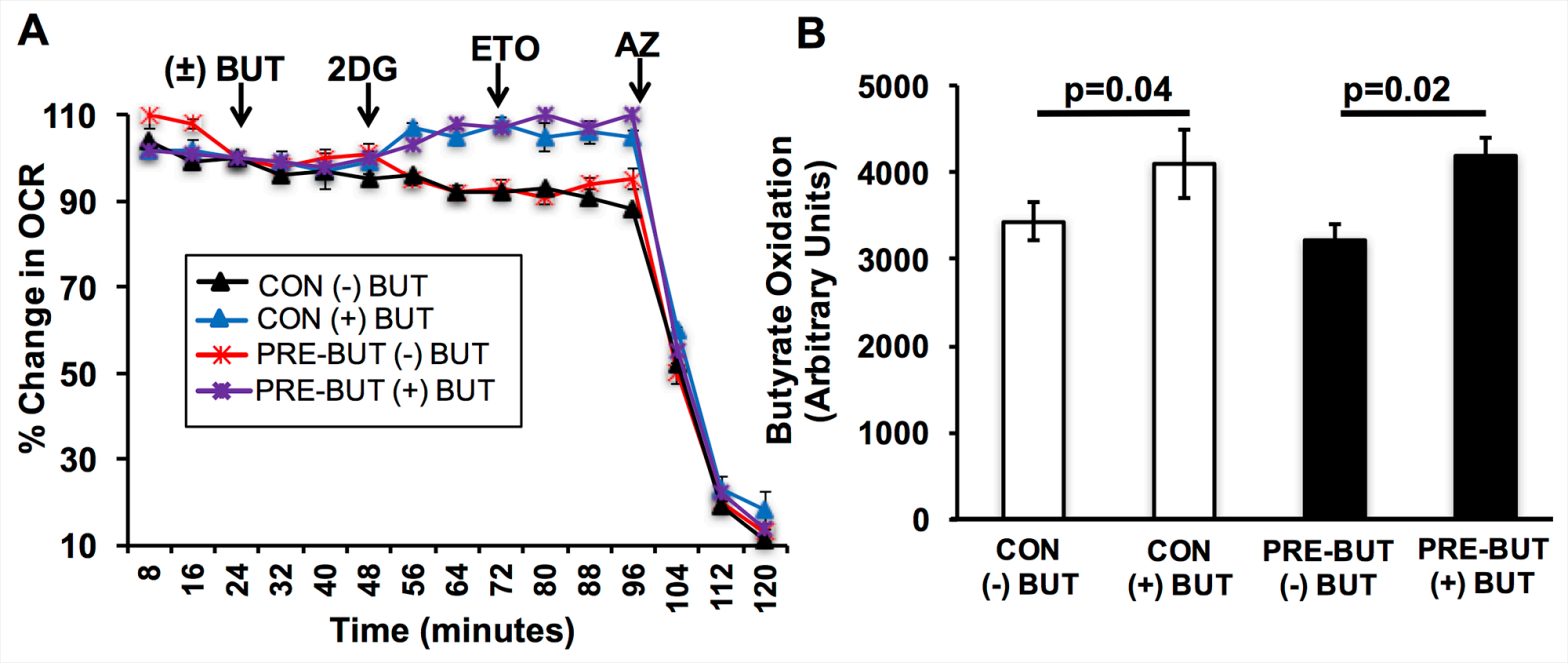
Butyrate has no effect on its oxidation in primary colonocytes **(A)** Percentage change in oxygen consumption rate (OCR) relative to baseline in isolated colonocytes treated with and without butyrate. **(B)** Area under the curve measurement from OCR analysis taken after 2-deoxyglucose (2DG) injection but before azide injection. Data for butyrate oxidation represent the average of 5 replicates per condition. Error bars are ± SEM. p<0.05 indicates significant difference between groups.

Non-cancerous colonocytes efficiently oxidize butyrate, and it is unclear whether pretreatment with butyrate would affect this process. In addressing this issue, colonocytes where isolated from adult mice, and pretreated with and without butyrate. Butyrate oxidation was measured as before in colonocytes after pretreatment. Butyrate increased the oxygen consumption rate to the same level after 2DG addition in both experimental conditions, non-pretreated and pretreated (Figure 2A). As opposed to colorectal cancer cells, pretreatment with butyrate did not show decreased oxidation when compared to non-pretreated colonocytes (Figure 2B). Thus, the ability of butyrate to decrease its own oxidation appears to be selective to colorectal cancer cells. Although, these future studies are needed to confirm this is the case in primary human colonocytes.

### Butyrate reduces SCAD expression only in colorectal cancer cells

**Figure 3:**
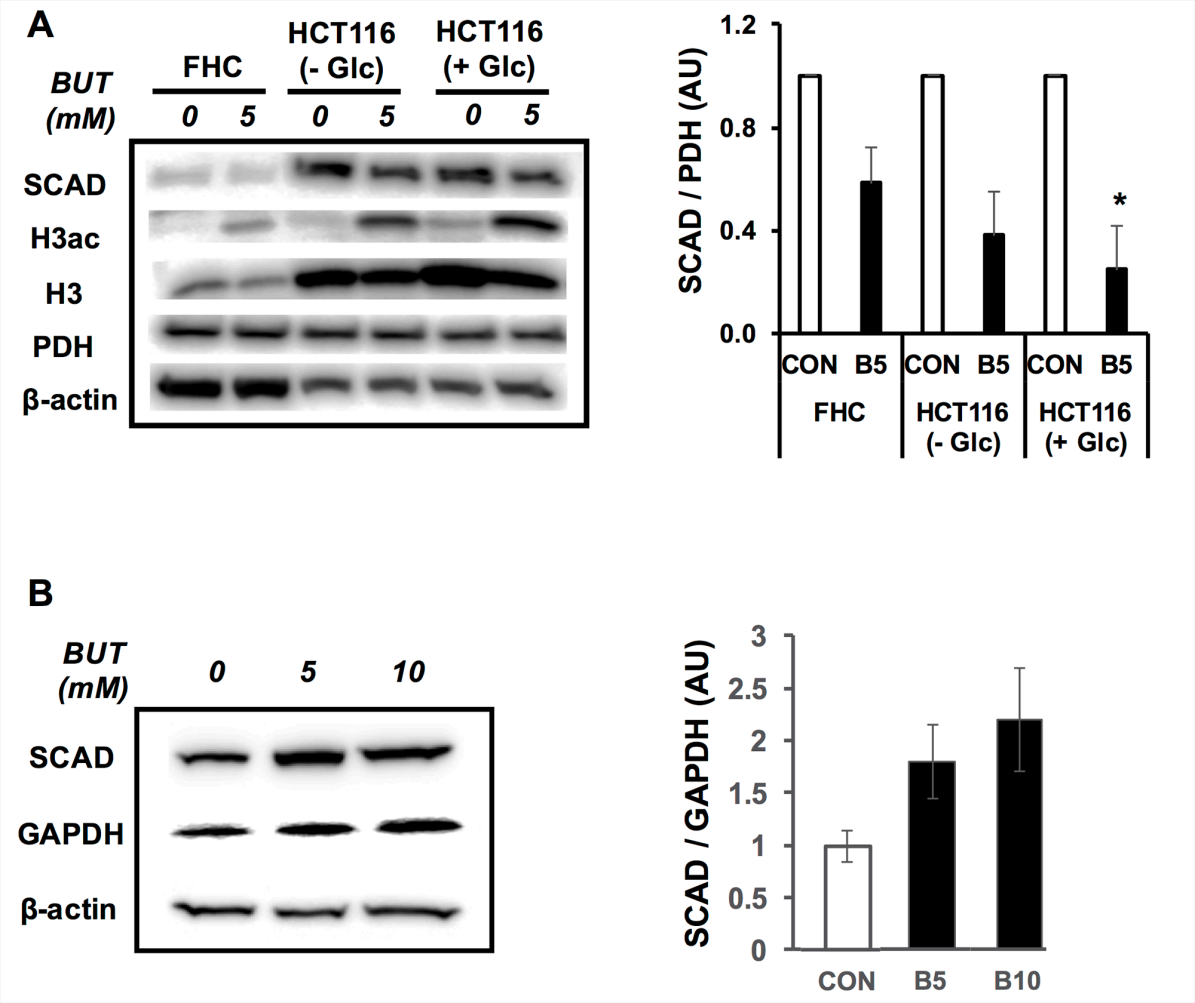
Butyrate decreases SCAD expression in cancerous but not primary colonocytes **(A)** Western blot showing SCAD expression in FHC cells and HCT116 cells that were treated with (0 mM, CON) or without butyrate (5 mM, B5). HCT116 cells were grown under the absence (2.5 mM glucose) and presence (25 mM glucose) for the Warburg effect. Right panel shows quantification of SCAD levels relative to PDH levels. **(B)** Western blot showing SCAD levels in isolated normal colonocytes with butyrate (0 mM, CON; 5 mM, B5 and 10 mM. B10). Quantification of SCAD expression relative to GAPDH is shown in right panel. For statistical analysis, western blot was conducted 5 times per condition. Error bars are Mean ± SEM. ^*^p<0.05 indicates significant difference between cells treated with butyrate vs. controls.

Butyrate decreased SCAD levels in colorectal cancer cells; however, it wasn’t clear whether butyrate would have this same effect in non-cancerous colonocytes? Cancerous colonocytes have a reduced ability to oxidize butyrate compared to non-cancerous colonocytes, which is in part due to lower cellular carnitine levels, and decreased organic cation/carnitine transporter (OCTN2) and carnitine palmitoyltransferase (CPT1A) [36]. In addition, the diminished ability of cancerous colonocytes to oxidize butyrate may be due to the lowered SCAD levels compared to the non-cancerous colonocytes. Therefore, we compared SCAD levels in cancerous (HCT116 cells) and non-cancerous (FHC cells). colonocyte lines. To our surprise, SCAD expression was actually higher in HCT116 cells than FHC cells (Figure 3A). This may be due to the fact that the expression and activity of enzymes related to fatty acid metabolism (i.e. SCAD) are dramatically elevated after birth in tissues where strongly metabolize fatty acids [37]. Since FHC cells originated from a 13-week fetus, these cells may not be a good representative cell line for non-cancerous colonocytes. However, Kaiko et al. (2011) confirmed that primary colonocytes (non-cancerous) have high SCAD expression compared to the stem cells in the colonic crypts [31].

As opposed to non-cancerous colonocytes, cancerous colonocytes increase aerobic glycolysis by utilizing the Warburg effect, which may alter the expression of proteins such as SCAD and negatively impact the oxidation of butyrate. To directly test the importance of the Warburg effect in butyrate-mediated SCAD reduction, the glucose concentration in the media was manipulated to high or low. SCAD expression was observed in the HCT116 cells under the high and low glucose conditions treated with butyrate (Figure 3A and Supplementary Figure 2). Importantly, SCAD levels were only significantly decreased under with the high glucose condition. To test whether butyrate decreases SCAD levels in cells not undergoing the Warburg effect, non-cancerous colonocytes were isolated from mice and treated with butyrate. Non-cancerous isolated colonocytes treated with butyrate showed a trend toward increasing SCAD levels, although this effect was not significant (Figure 3B). SCAD expression was normalized to GAPDH. Butyrate levels in the colonic lumen can range from 0.5mM to as high as 20mM in the colonic lumen [38–40]. Since no decrease in SCAD levels was observed at 5 mM butyrate, the concentration was increased to 10 mM, which is still within the physiological range. Nevertheless, there was no significant effect on SCAD expression even at 10 mM butyrate.

**Figure 4:**
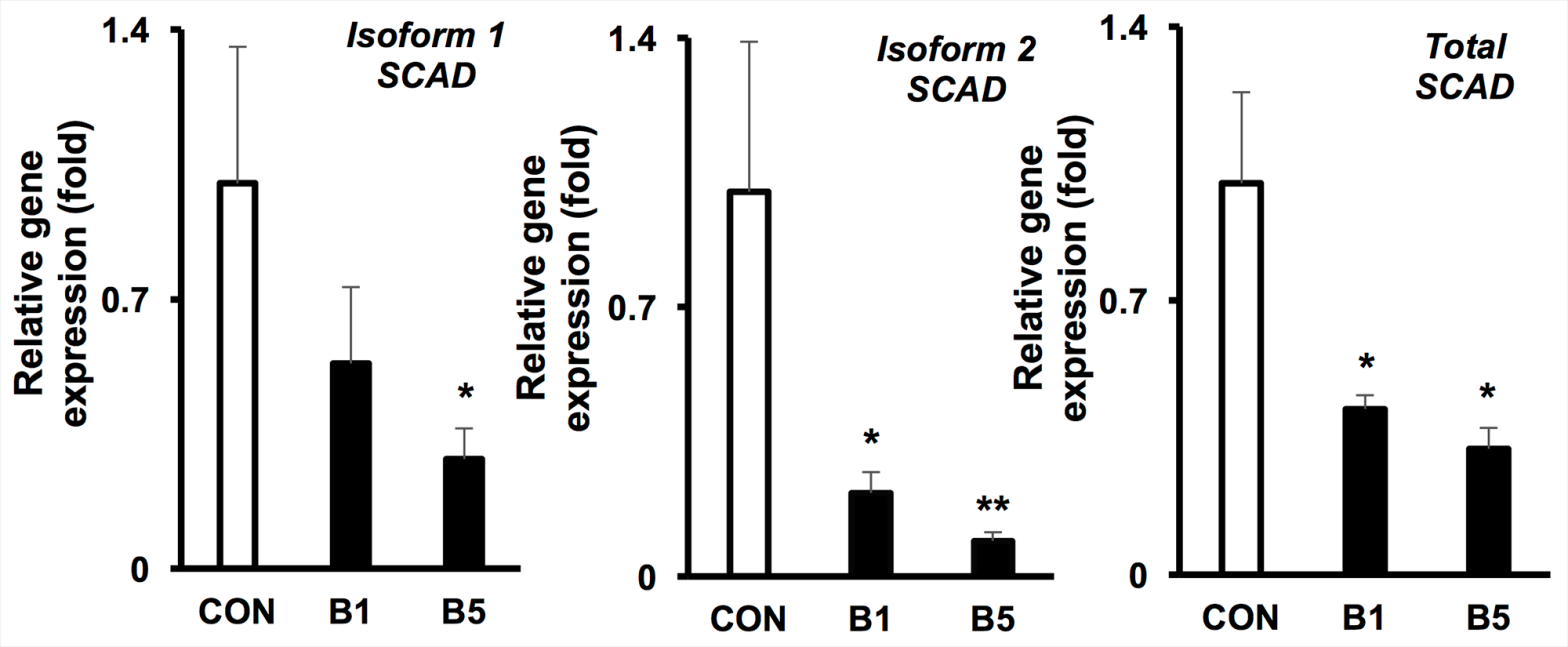
Butyrate reduces the mRNA levels of SCAD in colorectal cancer cell mRNA expression of *isoform 1*, *isoform 2* and total *Scad* was evaluated by semi-quantitative RT-PCR (0 mM, CON; 5 mM, B5 and 10 mM, B10). The relative mRNA level was normalized to *18S* rRNA and shown as fold of the control value. For statistical analysis, qRT-PCR was conducted three times per condition. Error bars are Mean ± SEM. ^*^p<0.05 and ^**^p<0.01 indicates significant difference between cells treated with butyrate vs. controls.

To confirm whether butyrate suppressed SCAD at the mRNA level, we conducted qRT-PCR and measured *Scad1*, *Scad2*, and total *Scad*. *Scad1* and *Scad2* are products of alternative splicing of the *Scad* gene. Butyrate, at 5 mM, significantly decreased mRNA levels for *Scad1*, *Scad2*, and total *Scad* in HCT116 colorectal cancer cells (Figure 4). Additionally, at a low butyrate concentration (1 mM), *Scad2* and total *Scad* mRNA were also significantly diminished. These data suggest that butyrate suppresses SCAD transcription and gene expression in colorectal cancer cells, which may involve inhibition of histone deacetylases.

### Butyrate lowers SCAD expression as an HDAC inhibitor

**Figure 5:**
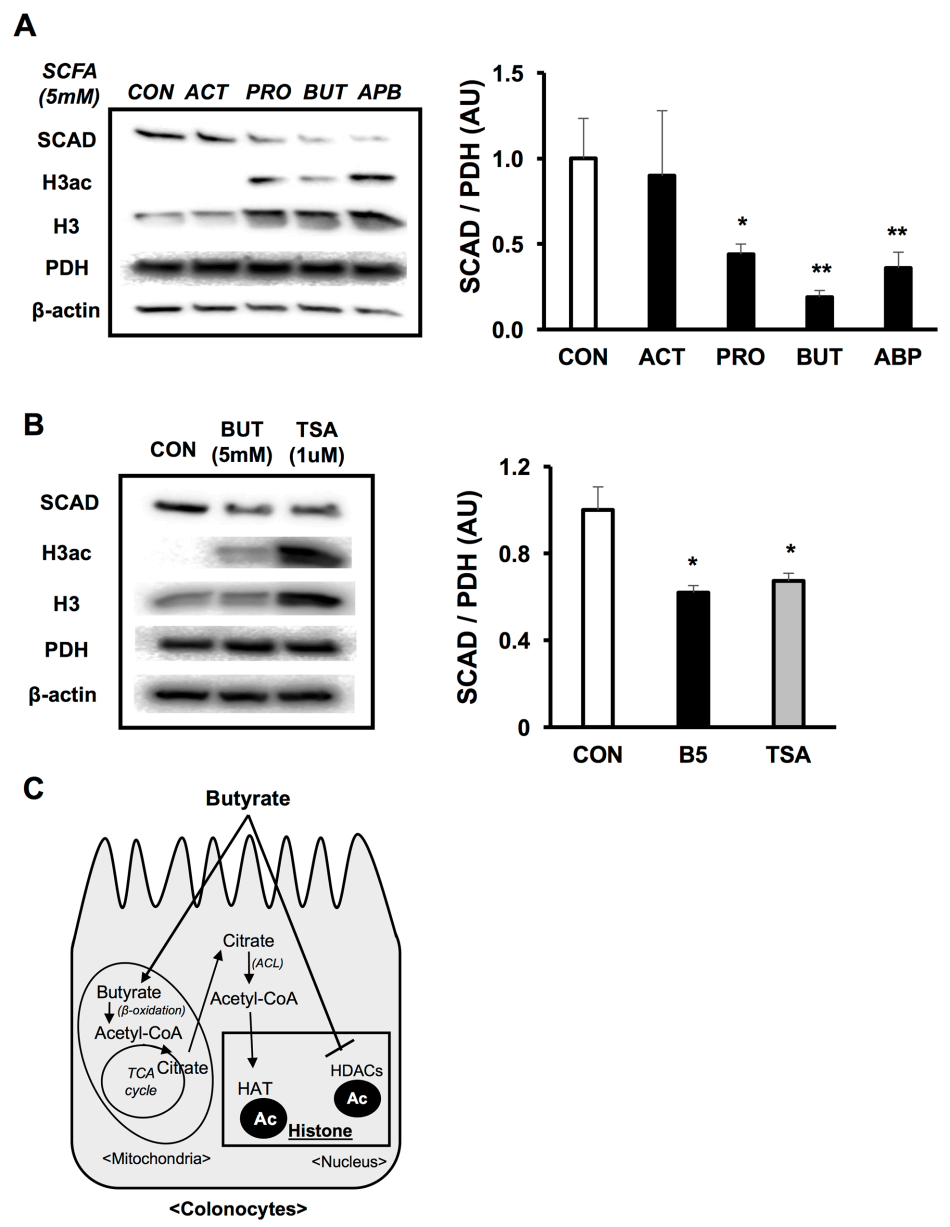
Butyrate decreases SCAD as an HDAC inhibitor **(A)** Western blot showing SCAD expression in HCT116 cells that were treated with a set of SCFAs (ACT; acetate, PRO; propionate, BUT; butyrate and APB; all of SCFAs) at 5 mM. Right panel shows quantification of SCAD levels relative to PDH levels. **(B)** Western blot describing SCAD expression in HCT116 cells that were treated with butyrate (5 mM) or TSA (1 μM). Quantification of SCAD expression relative to PDH levels is shown in right panel. **(C)** Diagram showing epigenetic mechanisms as to how butyrate can modulate gene expression. Butyrate acts as a co-factor for histone acetyltransferases (HATs) through the involvement of ATP-citrate lyase (ACL). Also, butyrate directly inhibits histone deacetylases (HDACs). For statistical analysis, western blot was conducted 3 times per condition. Error bars are Mean ± SEM. ^*^p<0.05 and ^**^p<0.01 indicates significant difference between cells treated with butyrate vs. controls.

The fermentation of dietary fiber in the colon produces additional SCFAs, which include acetate and propionate. Therefore, we wanted to investigate whether these other SCFAs reduced SCAD expression in colorectal cancer cells like butyrate. Both propionate and butyrate significantly decreased SCAD expression, while acetate did not impact SCAD expression in HCT116 colorectal cancer cells (Figure 5A). Similar to butyrate, propionate also inhibits histone deacetylases [41]. This alludes to HDAC inhibition as a key component in regulating SCAD expression in colorectal cancer cells.

Butyrate is involved in epigenetic modifications through two mechanisms (Figure 5C) [18]. Mitochondrial butyrate oxidation results in the biogenesis of acetyl-CoA. Acetyl-CoA can be utilized as a cofactor for histone acetyltransferases (HATs) when regenerated in the cytosol via ATP-citrate lyase (ACL). In addition, butyrate directly goes into the nucleus where it functions as an HDAC inhibitor. First, to test the importance of HDAC inhibition, a structurally distinct HDAC inhibitor, trichostatin A (TSA), was used as a positive control. Both butyrate and TSA significantly reduced SCAD expression (Figure 5B). Next, a siRNA knockdown of ACL was performed and SCAD expression was evaluated with and without butyrate. Since ACL catalyzes the reaction that converts citrate into acetyl-CoA in the cytosol, an ACL knockdown would block butyrate’s involvement as a HAT cofactor. However, knockdown of ACL did not impact SCAD suppression induced by butyrate, indicating that this mechanism is unrelated to SCAD regulation. Moreover, there was no difference in the percentage change in OCR between siMock and siACL transfected cells (Supplementary Figure 3A and 3B). In addition, butyrate still significantly reduced SCAD expression in both siMock and siACL knockdown, suggesting that ACL was not an important mediator in the decreased SCAD levels (Supplementary Figure 3C). Taken together, this data point to inhibition of histone deacetyases as the major mechanism as to how butyrate diminishes SCAD expression in the colorectal cancer cells.

### Butyrate decreases SCAD levels through selective inhibition of HDAC1

**Figure 6:**
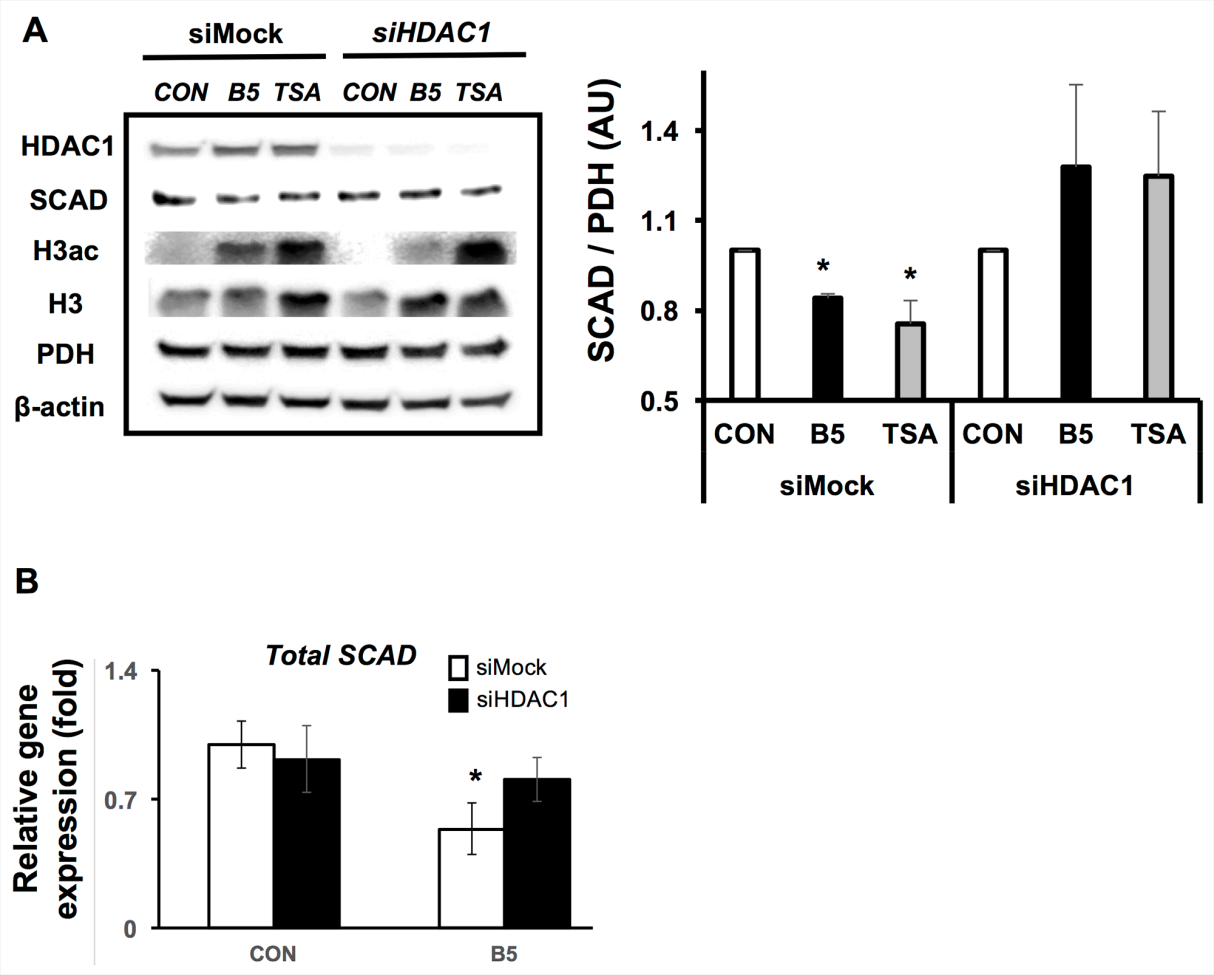
Butyrate inhibits HDAC 1 to reduce SCAD expression **(A)** Western blot showing SCAD expression for siMock and siHDAC1 transfected HCT116 cells after treatment with or without butyrate (5 mM) or TSA (1 μM) for 6hrs. Right panel shows quantification of SCAD levels relative to PDH levels. **(B)** mRNA expression of total *Scad* was evaluated by semi-quantitative RT-PCR. The relative mRNA level was normalized to 18S rRNA and shown as fold of the control value. For statistical analysis, qRT-PCR was conducted three times per condition. Error bars are Mean ± SEM. ^*^p<0.05 indicates significant difference between cells treated with butyrate vs. controls.

In colorectal cancer, HDAC 1, HDAC 2, and HDAC 3 are highly expressed in order to accelerate cell proliferation, growth and survival [42, 43]. Butyrate effectively inhibits most HDACs, which results in decreased cell proliferation and induction of cell apoptosis in the cancer cells [15, 18]. Since butyrate along with other HDAC inhibitors decreased SCAD levels, it was unclear whether a selective HDAC was responsible for the effect. Therefore, HDAC1, HDAC2, or HDAC3 were knocked down using RNAi and these cells were treated with butyrate to test whether SCAD reduction is augmented by a selective HDAC knockdown condition. While butyrate and TSA significantly decreased SCAD levels in siMock cells, siHDAC1 transfected cells did not show decreased SCAD levels after butyrate treatment (Figure 6A). *Scad* mRNA levels were also measured to confirm that knockdown of HDAC1 inhibited butyrate’s ability to reduce *Scad* gene expression. Butyrate was no longer able to decrease *Scad* mRNA levels when HDAC1 was knocked down (Figure 6B). RNAi knockdown of HDAC2 and HDAC3 in HCT116 cells did not impact SCAD levels like as HDAC1 (Supplementary Figure 4). These findings demonstrate that butyrate targets HDAC1 to decrease SCAD levels in the colorectal cancer cells. These data also suggest that although butyrate is a general HDAC inhibitor, its effects can be mediated through a specific HDAC.

## DISCUSSION

Normal colonocytes prefer to oxidize butyrate as a primary energy source whereas cancerous colonocytes increase glucose utilization. This is demonstrated through increased fluorodeoxyglucose (FDG) uptake as measured by positron emission tomography – computed tomography (PET-CT) in colorectal cancer patients [44–47]. In addition, colorectal cancer cells display a reduced capacity to oxidize butyrate compared to normal colonocytes, which could result from diminished intracellular carnitine and CPT1A levels [36]. These changes in metabolism significantly impact butyrate’s role as an inhibitor of histone deacetylases in colorectal cancer cells [18]. As an HDAC inhibitor, butyrate represses cell proliferation and induces cell death in cancer cells [16, 48, 49]. Thus, gaining knowledge toward the mechanisms that regulate butyrate oxidation in cancer cells is an important step in understanding butyrate’s role as an HDAC inhibitor, which is associated with the molecule’s protective action toward colorectal cancer. Here, it is demonstrated that butyrate suppresses its own oxidation in cancerous colonocytes. Butyrate diminishes SCAD levels, a critical enzyme involved in its oxidation. Specifically, butyrate regulates SCAD expression through inhibition of histone deacetylase 1 (HDAC1). The fact that butyrate increases histone acetylation, which is generally associated with upregulated gene expression might suggest that decreased SCAD levels observed after butyrate treatment most likely involves a secondary mechanism. An upregulation of a negative co-regulator of SCAD is a possibility.

Many studies have previously reported a role of SCAD in regulating butyrate oxidation [27, 28, 30]. SCAD contributes to energy metabolism in the colon via participating in SCFA oxidation [29]. Recently, *Kaiko et al.* reported that colonocytes isolated from SCAD^-/-^ mice showed diminished butyrate oxidation compared to those from normal mice (SCAD^+/+^), which confirms the importance of SCAD in mediating butyrate oxidation in colonocytes [31]. In contrast to this study, a cancerous colonocyte cell line was used due to previous reports demonstrating diminished butyrate oxidation in this cell line [18, 36]. The fact that butyrate reduces its own oxidation in these cells reveals a potential mechanism as to why colorectal cancer cells are sensitive to butyrate’s HDAC inhibitory effects.

This mechanistic relationship between butyrate and SCAD in the colorectal cancer cells was significantly influenced by the Warburg effect. Previously, it was found that butyrate (1 mM) increased SCAD expression in a colorectal cancer cell line (HT15). However, similar to a non-cancerous cell line, these cells still preferentially utilized butyrate over glucose thereby negating the impact of the Warburg effect [50]. The colonic administration of butyrate in healthy subjects increases gene transcription relating to energy metabolism and fatty acid metabolism, while SCAD was not altered [51]. Germfree mice, which lack a microbiome and the capability to produce butyrate from fiber, show reduced SCAD expression compared to normal mice [12]. These findings are consistent with butyrate modulating SCAD levels differently in non-cancerous colonocytes. However, more studies will be needed to confirm whether this holds true in non-cancerous human colonocytes since the primary colonocytes used in this study were from mice. Butyrate infusion studies in humans, which also reported no change in SCAD mRNA levels in colon [52].

In general, butyrate inhibits most HDACs, except class 2 (HDAC6 and 10) and class 3, and specifically inhibits HDAC1 and 3 in colorectal cancer cells [15, 53]. As an HDAC inhibitor, butyrate effectively impedes cancer cell survival and growth [15, 54]. Butyrate suppresses intestinal inflammation and oxidative stress, while protecting the intestinal epithelial barrier via its HDAC inhibitor role; these effects likely help reduce CRC susceptibility [55–57]. Separate from the non-cell autonomous effects, butyrate availability can influence cancerous colonocyte metabolism and HDAC inhibition. The suppressive action of butyrate on SCAD expression and its own oxidation in the colorectal cancer cells is mediated by its function as an HDAC inhibitor. Butyrate specifically inhibits HDAC1 to have these regulatory actions as knockdown of this protein negates any change in SCAD caused by butyrate. These data also allude to butyrate promoting its own action as an HDAC inhibitor in colorectal cancer cells through decreasing its own metabolism. It will be interesting to conduct further research regarding why butyrate behaves in this way, and whether this mechanism helps mediate its specificity toward cancer cells. Colorectal cancer (all stages) has altered gene expression characterized by lower mitochondrial metabolism and a down-regulation in SCAD expression [32, 33]. Future studies are needed to investigate the outcome of reduced SCAD expression in cancerous colonocytes especially as it relates to the Warburg effect and diminished butyrate oxidation.

The oncological significance of this work relates to the fact that colorectal cancer is, in part, a metabolic disease, where alterations in metabolic pathways are key to tumor development and progression. One important metabolic shift involves the cancer cell’s switch from utilizing butyrate as its primary energetic substrate to adopting a more glycolytic phenotype characterized by enhanced glucose uptake. This work provides insight into the mechanisms that impact this metabolic shift. As demonstrated that butyrate suppresses its own oxidation in colorectal cancer cells, which is associated with HDAC inhibition regulating SCAD. Reversing the colorectal cell’s glycolytic phenotype and forcing metabolism back toward butyrate utilization represents an unexplored therapeutic strategy.

## MATERIALS AND METHODS

### Cell culture and siRNA transfection

HCT116 cell (ATCC, CCL-247) were grown in DMEM supplemented with 25mM glucose and 10% FBS. FHC cells (ATCC, CRL-1831) were grown in complete growth DMEM:F12 medium following the recommended recipe from ATCC with 20% FBS instead of 10%. Specifically, the following reagents were added to the DMEM:F12 medium; HEPES (final concentration of 25 mM), 10 ng/mL of cholera toxin, 0.005 mg/mL of insulin, 0.005 mg/mL of transferrin, and 100 ng/mL of hydrocortisone. RNAi transfection in HCT116 cells was performed as previously described [18], and siRNA pools for human ACL (Dharmacon, #L-004915-00), human HDAC1 (Dharmacon, #L-003493-00-0005), human HDAC2 (Dharmacon, #L-003495-02-0005), human HDCA3 (Dharmacon, #L-003496-00-0005) and non-targeting control (Dharmacon, D001810-01-05) were used at a 20nM final concentration. The optimized time for each siRNA transfection was confirmed with Western blotting.

### Animal studies and colonocytes isolation

C57Bl/6J were purchased from Jackson Laboratories (Bar Harbor, ME) and were maintained on a 12-hour light-dark cycle with free access to water and standard laboratory chow (Purina lab chow, # 5001). Mouse studies were conducted according to federal regulations and were approved by the Institutional Animal Care and Use Committee at the University of Tennessee-Knoxville. Isolation of colonic epithelial cells from mice was performed from 8-12 weeks old male mice as previously described [12, 39]. Colons were washed several times with sterilized phosphate-buffered Saline (PBS). Then, the colon was incubated in PBS containing 5 mM EDTA (Fisher Scientific, Cat# S311-500) and 1% FBS, with or without butyrate (5 and 10mM), for 45 mins at 37°C. After 45 mins, the tissues were removed and isolated colonocytes were collected through centrifugation.

### Biochemical assays

HDAC activity assay was performed according to manufacturer specifications (BioVision, Cat# K339-100). Briefly, HCT116 cells were seeded into 96 well plates and treated with butyrate (Sigma, B5887) and trichostatin A (Promega, G6560). Following treatment times, assay was performed. All values were normalized to total protein in each well.

### Flux experiment

To measure percentage change of oxygen consumption rates (% OCR) in HCT116 cells, Seahorse XF^24^ Analyzer (Seahorse Bioscience) was used. All Seahorse assays were conducted according to the company guidelines, and the experimental design to measure butyrate oxidation in HCT116 cells were followed as stated [36]. HCT116 cells were seeded into XF^24^ cell culture microplates (Seahorse Bioscience, 100777-004) with an identical cell number per well. Cell plates are incubated with 1X KHB (2.5mM glucose and 50 μM carnitine) in non-CO_2_ incubator at 37°C for one hour before Seahorse assay. All Seahorse experiments were performed with identical conditions (unless otherwise stated). In brief, KHB media or sodium butyrate (Sigma, B5887) at 5mM final concentration were injected and the change in OCR was measured from baseline (% OCR). Then, 2-deoxyglucose (Sigma, D8375) was injected and % OCR was measured again. At last, 10% sodium azide was injected to block mitochondrial respiration by inhibiting complex IV.

### Western blot

From FHC, HCT116 cells and isolated colonocytes, the proteins are extracted with RIPA buffer (Cell Signaling, #9806), 1mM PMSF (Cell Signaling, #8553) and phosphatase inhibitor cocktail (Cell Signaling, #5872). Quantifications of protein were measured by Bradford assay. Gel electrophoresis and transfer were performed using standard protocol for Western blotting. Antibodies that were used included pan-acetylated-histone H3 (Active motif, Cat# 39139), total Histone H3 (Active motif, Cat# 39763), total PDH (Abcam, Cat# ab110330), ACL (Cell Signaling, Cat # 4332), SCAD (Abcam, Cat# 154823), HDAC1 (Cell signaling, Cat# 34589), HDAC2 (Cell signaling, Cat# 57156), HDAC3 (Cell signaling, Cat# 85057) and β-actin (Sigma, Cat# A1978). Chemiluminescence detection was conducted with the Odyssey Fc and bands were quantified with Image Studio Software (LI-COR Biosciences, Lincoln, NE).

### mRNA expression

Total RNA from (un) treated HCT116 cells were extracted using Trizol reagent (ambion, Cat# 15596-026). The concentration and integrity of RNA were measured by Nano-drop 1000. Reverse transcription was performed with RevertAid RT kit (Thermo Scientific, Cat# K1691) by following the company’s protocol. The amounts of product from RT-qPCR was measured by SYBR Green fluorescence (applied Biosystems, Cat# 4309155). SCAD primers for isoform 1 (Forward: GCGACTCATGGGTTCTGAAT and Reverse: TGCGACAGTCCTCAAAGATG), isoform 2 (Forward: GCCCGACTGGACCTATTTTT and Reverse: TGCGACAGTCCTCAAAGATG) and total (Forward: CAGGGATGGGCTTCAAGATA and Reverse: TGTCTGCCAACTTGAACTGG) were designed and their efficiency was confirmed by gel PCR. Relative gene expression levels were calculated through the ΔΔCt method and normalized to human 18S rRNA.

### Statistical analysis

For biochemical assays, Seahorse experiments, and western blotting, the differences between experimental groups were determined by ANOVA followed by a Tukey post-hoc test. All data are expressed as mean ± SEM. Groups are considered significantly different at p < 0.05.

## SUPPLEMENTARY MATERIALS FIGURES



## Abbreviations

HDAC: histone deacetylase
SCAD: acyl-CoA dehydrogenase – short chain
CRC: colorectal cancer
SCFAs: short-chain fatty acids
OCR: oxygen consumption rate
2DG: 2-deoxyglucose
MCT1: monocarboxylate transporter-1
OCTN2: organic cation/carnitine transporter
CPT1A: carnitine palmitoyltransferase-1A
FHC: fetal human colonocytes
PDH: pyruvate dehydrogenase
TSA: trichostatin A
ACL: ATP-citrate lyase
KHB: krebs-HEPES buffer
