# Supplementary material for: Butyrate decreases its own oxidation in colorectal cancer cells through inhibition of histone deacetylases
Source: Oncotarget. 2018 Jun 5;9(43):27280–92. doi: 10.18632/oncotarget.25546 (PMC6007476; doi:10.18632/oncotarget.25546)
Supplement: Supplementary file 1 [file oncotarget-09-27280-s001.pdf]

Butyrate decreases its own oxidation in colorectal cancer cells through inhibition of histone deacetylases

SUPPLEMENTARY MATERIALS

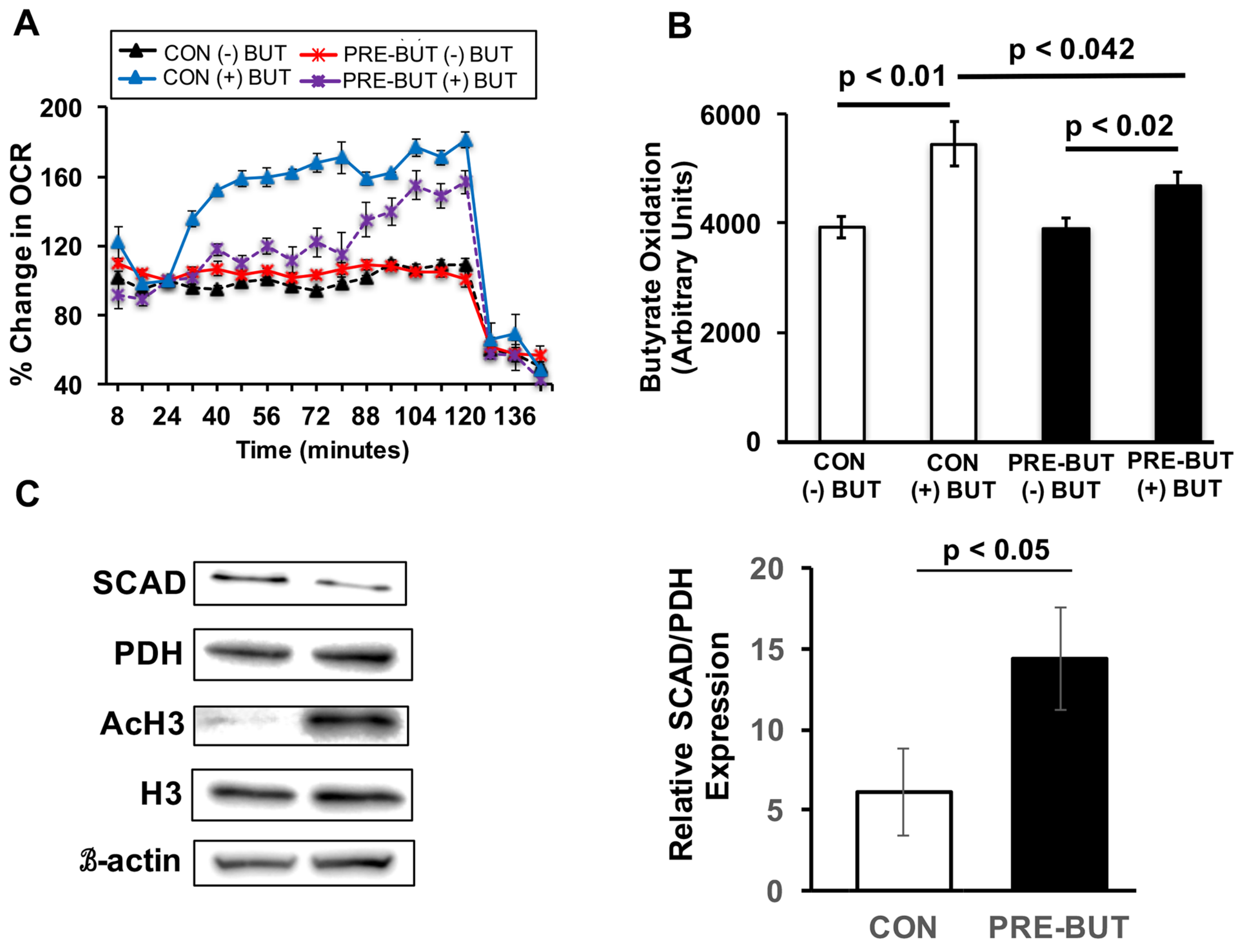

Supplementary Figure 1: Butyrate pretreatment suppresses oxidation in the RKO colorectal cancer cell line. **(A)** Percent change of oxygen consumption rate (OCR) relative to baseline in RKO colorectal cancer cells. OCR (%) after 2DG injection is total contribution of butyrate. **(B)** Area under the curve analysis from percentage change in OCR of in RKO cells with and without butyrate pretreatment. OCR measurement taken after 2DG injection, but before etomoxir (ETO) injection. **(C)** Western blot showing SCAD expression in RKO cells treated with butyrate. Quantification of SCAD levels relative to PDH levels. For statistical analysis, western blot was conducted three times per condition. Error bars are ± SEM.

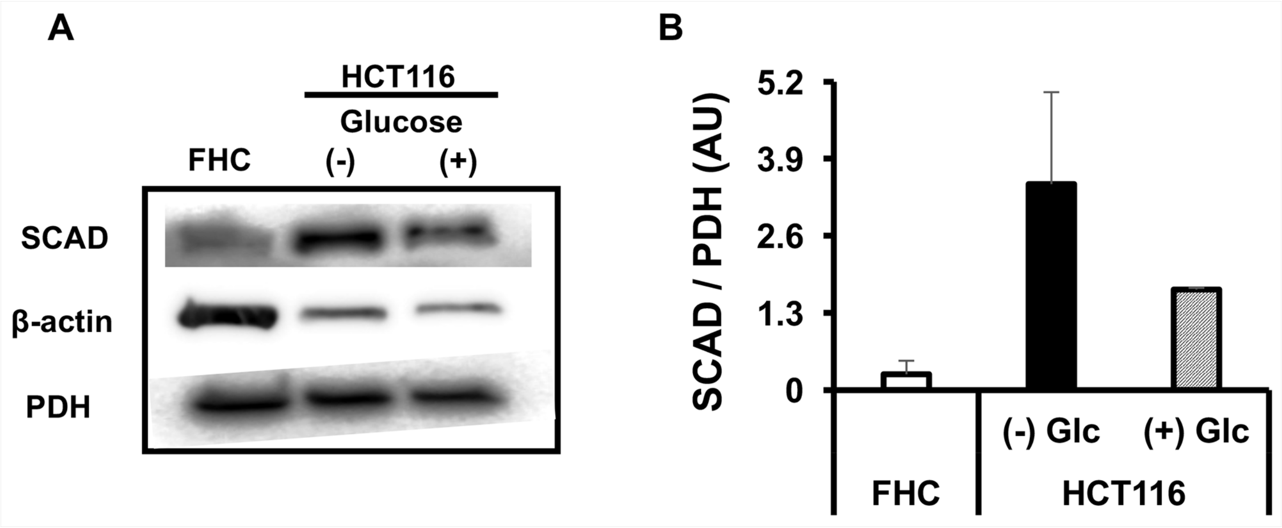

Supplementary Figure 2: The Warburg effect alters SCAD levels cancer cells. **(A)** Western blot showing SCAD expression in FHC cells (non-cancerous colonocytes) and HCT116 cells (colorectal cancer cells) with or without Warburg effect. **(B)** Quantification of SCAD levels relative to PDH levels. For statistical analysis, western blot was conducted three times per condition. Error bars are ± SEM.

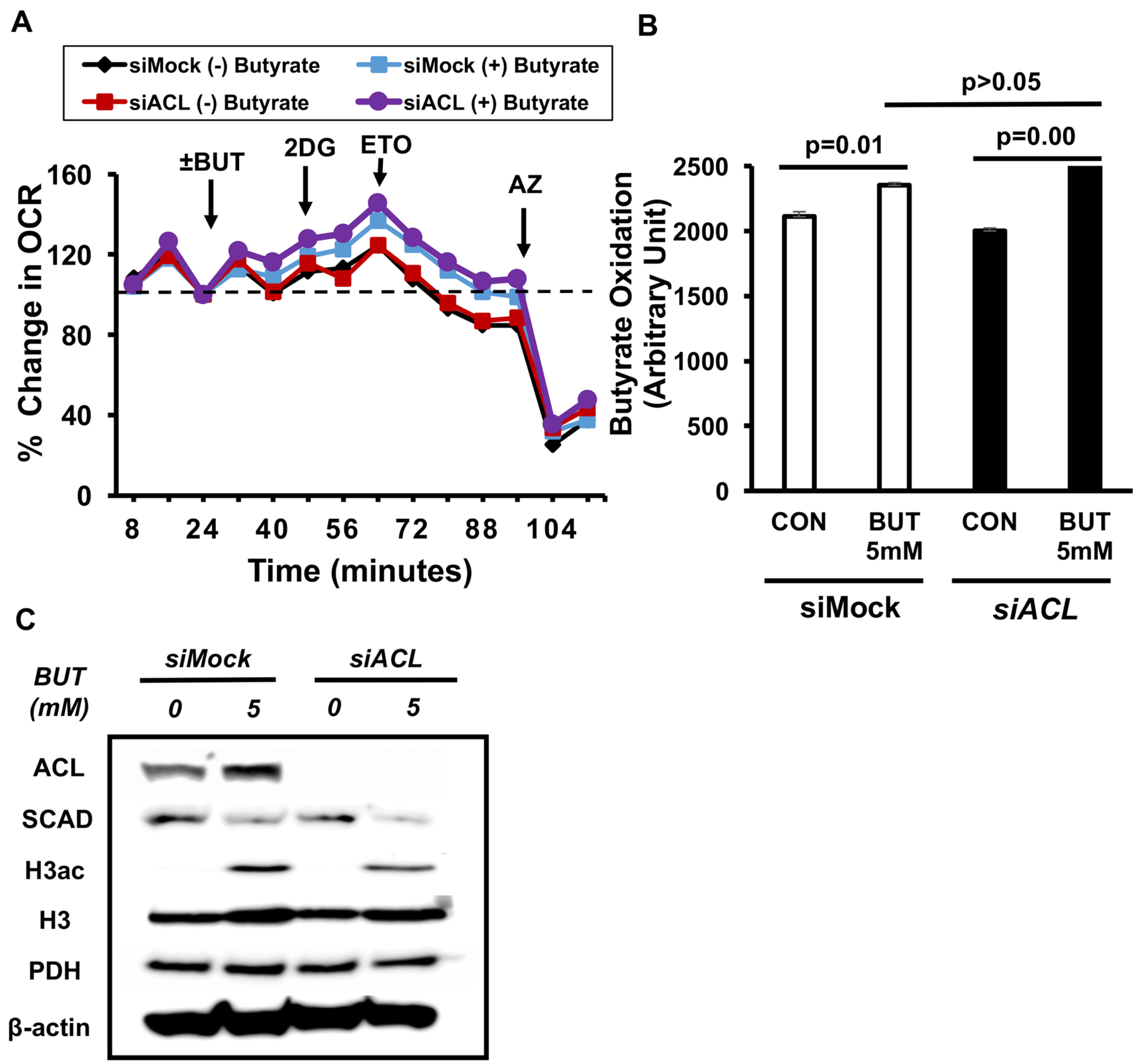

Supplementary Figure 3: Butyrate does not regulate SCAD expression as a metabolite in colorectal cancer cell. (A) Percent change of oxygen consumption rate (OCR) relative to baseline in siACL and siMock transfected HCT116 cells. OCR (%) after 2DG injection is total contribution of butyrate. **(B)** Area under the curve analysis from percentage change in OCR of siMock and siACL transfected HCT116 cells with and without butyrate (5 mM). OCR measurement taken after 2DG injection, but before etomoxir (ETO) injection (48-72 mins) **(C)** Western blot presenting SCAD levels siMock and siACL transfected HCT116 cells after treatment of butyrate or not. Data for butyrate oxidation measurement represent the average OCR (%) over 3-5 replicates per condition. Error bars are ± SEM. For statistical analysis, western blot was conducted three times per condition. Error bars are ± SEM.

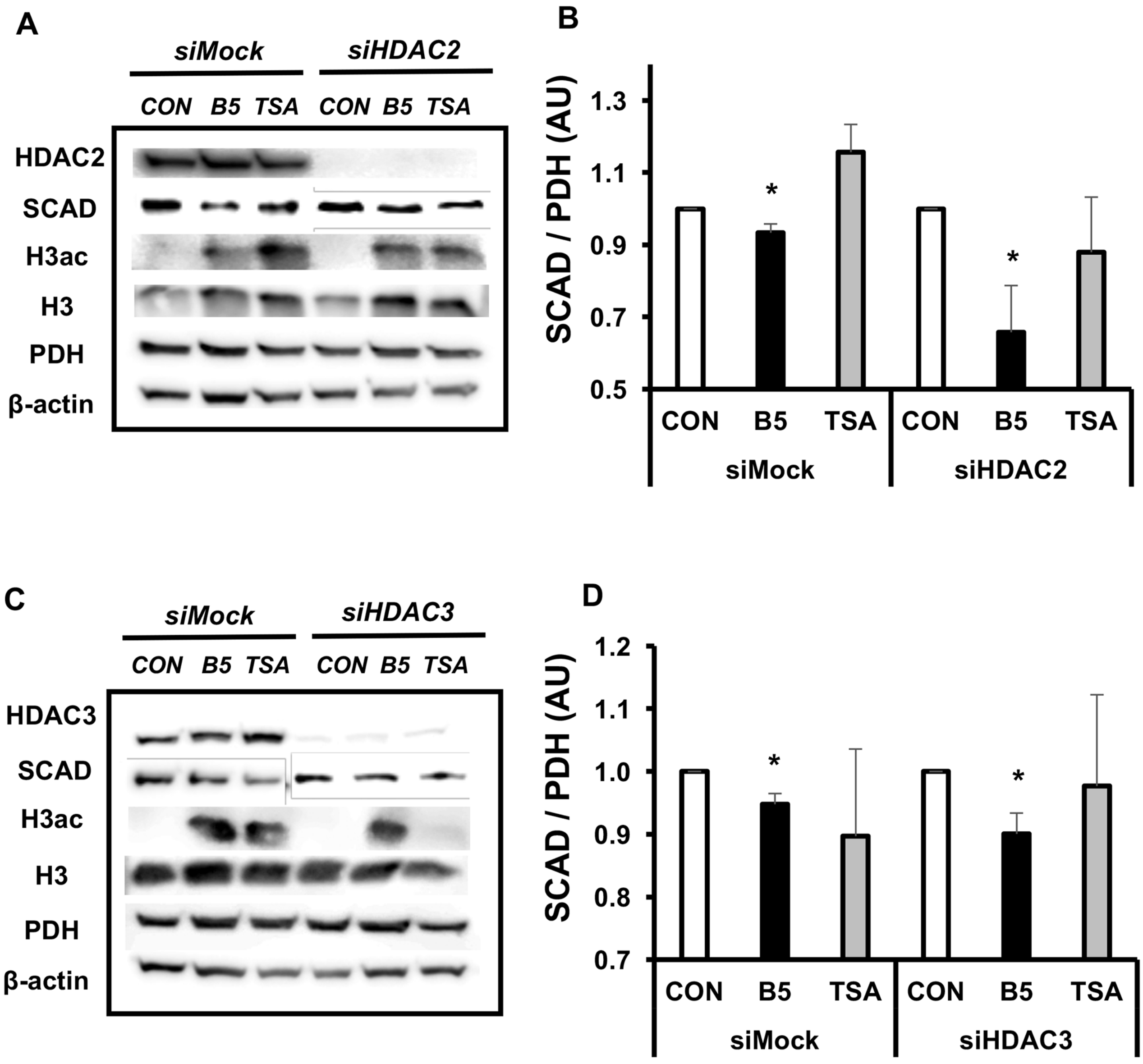

Supplementary Figure 4: HDAC 2 and HDAC 3 are not involved in SCAD suppression by butyrate. **(A)** Western blot presenting SCAD levels in siMock and siHDAC2 transfected HCT116 cells after treatment without butyrate and with butyrate (5 mM) or TSA (1 μM) for 6 hrs. **(B)** Quantification of SCAD levels relative to PDH levels. **(C)** Western blot showing SCAD levels in siMock and siHDAC3 transfected HCT116 cells after pre-treatment without butyrate and with butyrate (5 mM) or TSA (1 μM). **(D)** Quantification of SCAD levels relative to PDH levels. For statistical analysis, western blot was conducted three times per condition. Error bars are ± SEM. *$p<0.05$ indicates significant difference between cells treated with butyrate vs controls.
